# Integration of single-cell and bulk RNA sequencing to identify unique tumor stem cells and construct novel prognostic markers for assessing ESCA prognosis and drug sensitivity

**DOI:** 10.3389/fonc.2025.1649877

**Published:** 2025-08-27

**Authors:** Jia Shi, Danni Qiao, Qiongyang Lv, Yaliang Fan, Haibin Yu, Guiming Hu, Longhao Wang, Beibei Sha

**Affiliations:** ^1^ Department of Pathology, The Second Affiliated Hospital of Zhengzhou University, Zhengzhou, China; ^2^ Department of Interventional, The Second Affiliated Hospital of Zhengzhou University, Zhengzhou, China; ^3^ Department of Oncology, Henan Provincial People’s Hospital, People’s Hospital of Zhengzhou University, Zhengzhou, Henan, China

**Keywords:** single-cell RNA, gene signature, prognostic model, esca, tumor stem cell

## Abstract

**Background:**

Cancer stem cells (CSCs) are crucial contributors to the development and progression of esophageal cancer (ESCA). This study utilized single-cell RNA sequencing (scRNA-seq) and bulk RNA sequencing (RNA-seq) to identify gene signatures of CSCs in ESCA, aiming to construct a prognostic tumor stem cell marker signature (TSCMS) model.

**Methods:**

We analyzed scRNA-seq and RNA-seq data of ESCA. CytoTRACE was used to quantify the stemness of tumor-derived epithelial cell clusters. The TSCMS model was developed using Lasso-Cox regression, and its prognostic significance was evaluated via Kaplan-Meier survival analysis, Cox regression, and ROC curve analysis. Drug response predictions were conducted using the pRRophetic package. Functional studies of TSPO in ESCA cells included bioinformatics analysis, quantitative reverse transcription PCR (qRT-PCR), Western blotting, immunohistochemistry, and cell proliferation assays.

**Results:**

Distinct cell cluster stemness potentials were identified using CytoTRACE. The TSCMS model consists of 18 tumor stemness-related genes. High-risk patients showed reduced immune and ESTIMATE scores, along with elevated tumor purity. Notable differences in immune infiltration and chemotherapy sensitivity were observed between risk groups. TSPO was found to be positively correlated with RNA expression-based stemness scores in various tumors, including ESCA. Its expression was diminished in ESCA cell lines and clinical tumor tissues, with low expression correlating with poor prognosis. Overexpression of TSPO inhibits the proliferation of ESCA cells and the formation of tumor clones. In a mouse model of esophageal carcinoma *in situ*, TSPO expression was significantly lower than in normal tissues.

**Conclusion:**

This study underscores the prognostic significance of the TSCMS model in ESCA, elucidates the immune landscape and treatment response, and identifies TSPO as a potential therapeutic target.

## Introduction

Esophageal cancer (ESCA) is a highly aggressive malignancy and ranks among the leading causes of cancer-related deaths worldwide (1). It primarily consists of two major histological subtypes: esophageal squamous cell carcinoma (ESCC) and esophageal adenocarcinoma (EAC), each characterized by distinct epidemiological and molecular features (1–3). Despite advancements in surgical techniques, chemotherapy, radiotherapy, and immunotherapy, the prognosis for ESCA remains poor, with a five-year survival rate of less than 20% in most regions (4). A significant obstacle to improving clinical outcomes is the considerable intratumoral heterogeneity and the limited understanding of the interactions between tumor cells and their surrounding microenvironment (5). Traditional bulk RNA sequencing methods average gene expression across thousands of cells, potentially obscuring critical biological signals from rare or functionally distinct subpopulations (6). In recent years, single-cell RNA sequencing (scRNA-seq) has revolutionized transcriptomic analysis by enabling high-resolution profiling of individual cells and uncovering the complex cellular architecture of tumors (7, 8). The application of scRNA-seq to ESCA holds great promise for unveiling novel cellular phenotypes, elucidating lineage hierarchies, and identifying potential therapeutic targets (9).

Recent studies have underscored the pivotal role of cancer cell stemness in driving tumorigenesis, progression, therapeutic resistance, and recurrence across various malignancies, including esophageal cancer (10). Cancer stem-like cells (CSCs), defined by their self-renewal, multipotent differentiation potential, and high tumorigenicity, have been identified in both esophageal squamous cell carcinoma (ESCC) and esophageal adenocarcinoma (EAC) (11, 12). These cells are believed to contribute to tumor heterogeneity and plasticity, thereby facilitating adaptation to adverse microenvironments and evasion of cytotoxic therapies (13). Furthermore, transcriptional programs associated with stem cell properties, such as those regulated by SOX2, OCT4, and NANOG, have been implicated in sustaining the cancer stem cell population and promoting aggressive tumor phenotypes (14). However, a comprehensive understanding of CSC distribution, phenotypic transitions, and functional interactions within ESCA and its surrounding microenvironment remains limited, largely due to the resolution limitations of bulk analysis approaches (15). The advent of single-cell transcriptomic technologies provides a powerful approach to dissect these stemness-related characteristics at single-cell resolution, enabling the identification of rare CSC populations and revealing dynamic changes in stemness features during disease progression or treatment (16).

In the pursuit of accurate predictions, numerous studies have focused on developing prognostic models. Publicly available datasets such as TCGA and GEO provide extensive esophageal cancer samples with corresponding clinical information, enabling the development and robust validation of prognostic models. In this study, we utilized single-cell RNA sequencing to investigate cancer stem cells in esophageal cancer, identify key stemness-associated gene signatures, and construct a prognostic risk model. Our analysis led to the identification of a tumor stem cell marker signature (TSCMS) capable of predicting patient outcomes and informing therapeutic strategies. Furthermore, we identified the mitochondrial transporter protein (TSPO) as a key oncogene associated with ESCA stemness and poor prognosis, indicating its potential as a therapeutic target. These findings underscore the potential of the TSCMS model in enhancing prognosis and personalizing treatment strategies for esophageal cancer patients.

## Materials and methods

### Data collection

Single-cell RNA sequencing (scRNA-seq) and bulk RNA sequencing data were retrieved from the Gene Expression Omnibus (GEO) and The Cancer Genome Atlas (TCGA) databases. The single-cell dataset (GSE188900) from GEO comprises one normal sample and six esophageal cancer (ESCA) samples. The bulk RNA sequencing data were downloaded from the TCGA-ESCA cohort, comprising a total of 181 samples. Additionally, data for drug IC50 prediction were derived from a statistical study. All datasets utilized in this research were obtained from public databases or shared by other researchers.

### Preprocessing of scRNA−seq data

We imported the raw expression matrix using the R package Seurat and filtered the data to retain single-cell profiles from esophageal cancer (ESCA) and normal tissues. Cells with mitochondrial gene content exceeding 30% or expressing more than 10,000 genes were excluded from the analysis. For normalization, we utilized the SCTransform function, which mitigates technical noise and ensures uniform scaling across cells. Following this, we applied the RunPCA function with the parameter set to npcs = 50, and the RunUMAP function with parameters set to reduction = “pca” and dims = 1:20. Finally, we performed clustering by applying the FindNeighbors function (using PCA reduction with dims = 1:20) followed by the FindClusters function with a resolution of 0.1, resulting in the identification of 12 distinct cell clusters.

### Annotation of cellular subpopulations

After obtaining 16 clusters, we annotated these clusters with cell types based on the expression of specific marker genes (27). Immune cells were identified using marker genes such as PTPRC, with specific subsets including B cells (CD19, CD79A, IGKC, JCHAIN, MS4A1, MZB1), cancer stem cells (SOX9, CD44), and T cells (PTPRC, CD3D, CD3E, CD4, CD8A). Non-immune cell populations were also characterized, including epithelial cells (EPCAM, JUP, KRT14, KRT5, PPL), endothelial cells (CDH5, ENG, PECAM1, RAMP2, VWF), and fibroblasts (COL1A1, COL1A2, COL3A1, COL6A1, DCN, FN1). This classification resulted in the identification of 10 major cell types within the dataset.

### Differential gene analysis

In single-cell RNA sequencing (scRNA-seq) analysis, the FindAllMarkers function in the Seurat package was used to identify highly expressed genes, with parameters set to only.pos = TRUE and logfc.threshold = 0.25, while all other settings were kept at default. Differential gene expression analysis results for epithelial cell clusters in scRNA-seq are shown in **Supplementary Table S1** and visualized using the EnhancedVolcano package in R. For bulk RNA sequencing differential analysis, the DESeq2 package was applied with default settings. The samples were categorized into high-risk and low-risk groups based on the median for the differential analysis, and the results of the differentially expressed genes between these groups are displayed in **Supplementary Table S1**.

### Cellular stemness

CytoTRACE predicts cellular stemness at the single-cell level by leveraging gene expression data and intrinsic stemness gene sets. To identify tumor epithelial cell clusters with the highest stemness or lowest differentiation, we applied the CytoTRACE pipeline in R. Stemness-associated genes with correlation coefficients greater than 0.3 are listed in **Supplementary Table S2**.

### Gene functional enrichment analysis

Gene Set Enrichment Analysis (GSEA) of the TSCMS model was performed using the R package fgsea with default settings. Differentially expressed genes between high- and low-risk groups in the TCGA training set were ranked by fold change. KEGG pathway enrichment results are provided in **Supplementary Table S3**. Additionally, Gene Ontology (GO) enrichment analysis was conducted to evaluate the metabolic functions of these differentially expressed genes.

### Construction and validation of the prognostic risk model TSCMS

By intersecting dry-related genes with the differentially expressed genes from tumor epithelial cell clusters, we conducted univariate Cox regression analysis to evaluate their prognostic significance for overall survival in ESCA patients from the TCGA dataset. Genes with p-values below 0.05 were considered prognostic candidates. These candidate genes were then subjected to Least Absolute Shrinkage and Selection Operator (LASSO) Cox regression using the ‘glmnet’ package. Using ten-fold cross-validation, we selected genes with non-zero coefficients from the optimal feature selection. The final risk model was constructed as a linear combination of gene expression levels weighted by their corresponding risk coefficients. Patients were divided into low- and high-risk groups according to the median risk score. To validate the prognostic performance of the TSCMS model, we calculated the area under the curve (AUC) with the ‘timeROC’ package and performed Kaplan–Meier survival analysis. Statistical significance between groups was assessed using the log-rank test implemented in the ‘survminer’ R package.

### Drug response prediction

We utilized the pRRophetic package to conduct drug response prediction. Gene expression profiles of the high- and low-risk groups were used to estimate IC50 values for a range of commonly used clinical and preclinical antitumor drugs. Statistical analyses identified drugs with significantly different IC50 values between the two risk groups (**Supplementary Table S4**).

### Animal experimentation

Male C57BL/6 mice aged 6 to 8 weeks were acquired from Cyagen (China) and housed under specific pathogen-free (SPF) conditions, with unrestricted access to food and water. To induce esophageal carcinogenesis, the mice were administered 4-nitroquinoline 1-oxide (4NQO, Sigma-Aldrich) in their drinking water at a concentration of 100 μg/mL for a duration of 16 weeks, followed by a subsequent 8-week period of normal water. Control mice were provided only with regular water. At week 24, mice were euthanized, and esophageal tissues were collected, fixed in 10% formalin, paraffin-embedded, sectioned, and stained with hematoxylin and eosin (H&E). Histopathological alterations were assessed by two blinded pathologists and categorized as hyperplasia, dysplasia, carcinoma *in situ*, or squamous cell carcinoma.

### Immunohistochemistry

In this study, all paraffin-embedded tissues examined were normal esophageal tissues and induced carcinoma *in situ* tissues obtained from mice. For immunohistochemical staining, sections that had been deparaffinized and rehydrated were subjected to antigen retrieval by boiling in sodium citrate buffer (10 mM, pH 6.0) for 30 minutes. Sections were incubated with primary antibodies and developed using the Ultra Vision detection system. Images were acquired with an Olympus IX51 microscope and processed using cellSens Dimension software. The histochemical score (H-Score) was calculated using the formula ∑ (pi × i), where i represents the intensity grading of positive cells: 0 for negative (no staining); 1 for weakly positive (light yellow); 2 for moderately positive (brownish yellow); and 3 for strongly positive (brown). Here, pi denotes the percentage of cells at each corresponding intensity level. This formula can be expressed as: H-Score = (percentage of weakly positive cells × 1) + (percentage of moderately positive cells × 2) + (percentage of strongly positive cells × 3). The H-Score ranges from 0 to 300, with higher scores indicating stronger overall positivity by integrating both staining intensity and the proportion of positive cells.

### RNA isolation and quantitative real-time polymerase chain reaction

Total RNA was extracted from lung cancer cells and tissues using TRIzol reagent (Invitrogen Life Technologies). Real-time quantitative PCR was performed in an Agilent Mx3005P using SYBR qPCR Mix (MQ10201s, Monad). The samples were amplified under the following conditions: 40 cycles of 95°C/30 s, 95°C/5 s, and 60°C/30 s. The mRNA abundance of each gene of interest was normalized to that of β-actin. Data were analyzed by 2−ΔΔCt. Details of the primer sequences were as follows: TSPO forward 5′-GGGCCTTGGTGGATCTCCT-3′and reverse 5-TGTCCCGCCATACGCAGT-3′; GAPDH forward 5′-ATCATCAGCAATGCCTCC-3′and reverse 5′-CATCACGCCACAGTTTCC-G-3′.

### Cell culture

Three human esophageal squamous cell carcinoma (ESCC) cell lines, HET-1A, KYSE30, KYSE450, and EC1, were used in this study. All cell lines were purchased from the Shanghai Institute of Biochemistry and Cell Biology, Chinese Academy of Sciences (Shanghai, China). KYSE30 and KYSE450 cells were cultured in RPMI-1640 medium (Solarbio, China), and EC1 cells were maintained in DMEM medium (Solarbio, China), both supplemented with 10% fetal bovine serum (FBS; Solarbio, China), 100 U/mL penicillin, and 100 µg/mL streptomycin. Cells were cultured at 37 °C in a humidified incubator containing 5% CO_2_ and were routinely passaged every 2–3 days.

### Colony formation assay

For the colony formation assay, ESCC cells (KYSE30, KYSE450, and EC1) were seeded in 6-well plates at a density of 500 cells per well, depending on the growth rate of each cell line. After incubation for 10 days under standard culture conditions (37 °C, 5% CO_2_), colonies were fixed with 4% paraformaldehyde for 15 minutes and stained with 0.1% crystal violet for 20 minutes. Visible colonies (consisting of >50 cells) were photographed and counted manually or using ImageJ software. All experiments were performed in triplicate.

### Cell viability assay

Cell viability was assessed using the Cell Counting Kit-8 (CCK-8; beyotime, China) according to the manufacturer’s instructions. ESCC cells (KYSE30, KYSE450, and EC1) were seeded into 96-well plates at a density of 2×10³ cells per well in 100 μL of complete medium. After incubation for 0, 24, 48, and 72 hours, 10 μL of CCK-8 reagent was added to each well, followed by incubation at 37 °C for 1–2 hours. The absorbance at 450 nm was measured using a microplate reader (Bio-Rad, USA). Each condition was tested in triplicate, and the experiments were independently repeated at least three times.

### Statistical analysis

Depending on data distribution and normality assumptions, differences between groups were assessed using either Student’s t-test or the Wilcoxon rank-sum test. Survival differences were evaluated by the Log-Rank test. A p-value < 0.05 was considered statistically significant. The levels of statistical significance are denoted as follows: * indicates *p* < 0.05, ** indicates *p* < 0.01, *** indicates *p* < 0.001, **** indicates *p* < 0.0001.

## Results

### The landscape of cell types in ESCA and normal tissues

**Figure 1:**
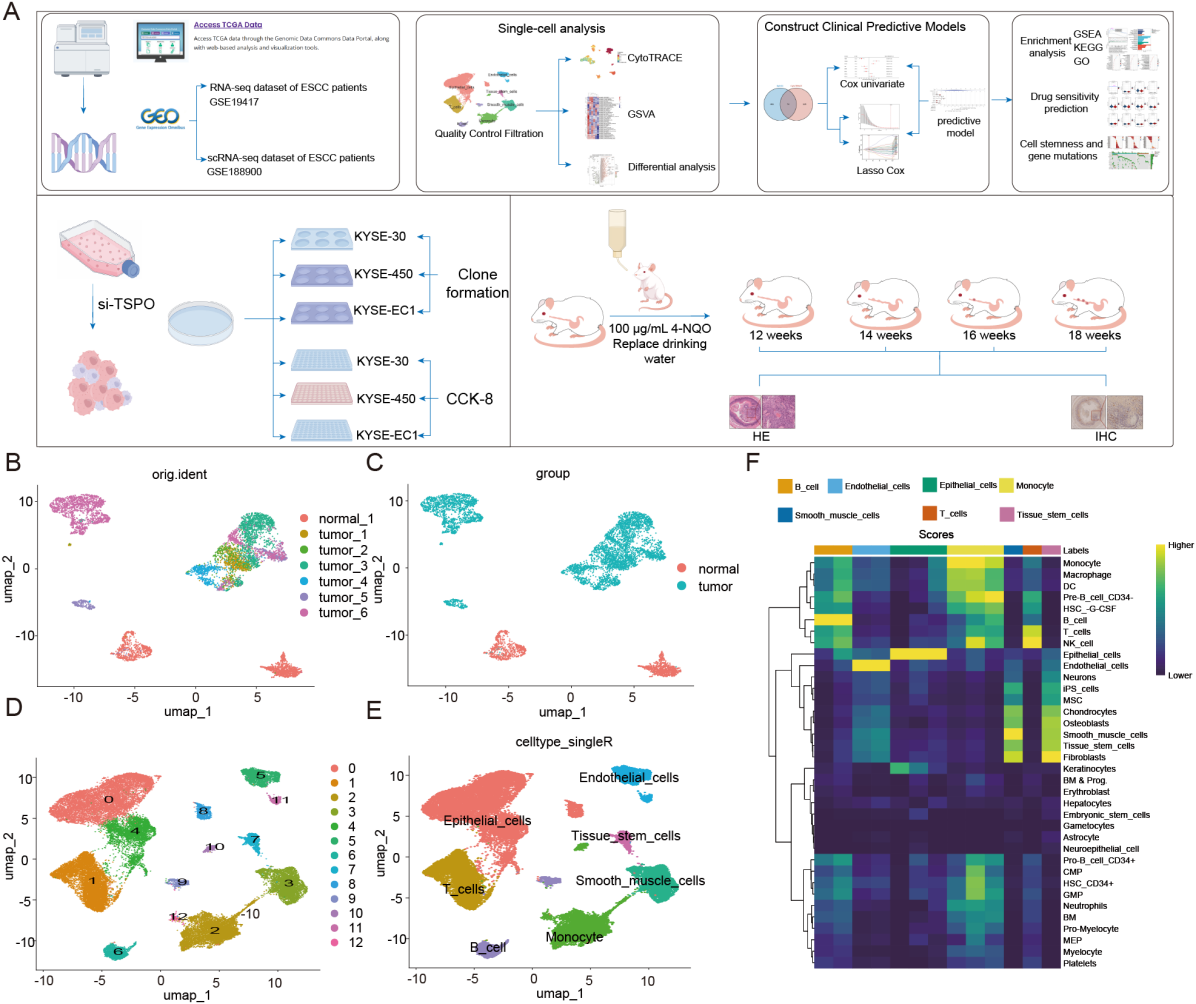
The landscape of cell types in ESCA and normal tissues. **(A)** Workflow of this study. **(B)** UMAP plot showing the distribution of cells divided by original identity (orig.ident), including one normal sample and six tumor ESCC samples. **(C)** UMAP plot colored by group (normal vs. tumor), showing clear separation between tumor samples and normal samples. **(D)** UMAP plot displaying unsupervised clustering based on gene expression, dividing cells into 13 clusters. **(E)** UMAP plot presenting cell types predicted using SingleR annotation, including epithelial cells, T cells, B cells, monocytes, tissue stem cells, smooth muscle cells, and endothelial cells. **(F)** Use reference marker features to display a heatmap of cell type enrichment scores across clusters, further validating cell identity annotations. The color intensity indicates the level of enrichment scores.

To investigate the potential functions of cancer stem cells in esophageal carcinoma, we analyzed bulk RNA-seq data from TCGA-ESCA and GEO (GSE53625), alongside single-cell RNA-seq data from GEO (GSE188900). Stemness scores of tumor epithelial cells were predicted using single-cell RNA sequencing (scRNA-seq) analysis. Subsequently, we constructed a prognostic model for esophageal carcinoma (ESCA) based on cancer stem cell genes and further validated its predictive ability (**Figure 1A**). Additionally, we conducted enrichment analyses and drug sensitivity predictions for both high-risk and low-risk groups to investigate the metabolic differences and identify highly sensitive drugs between these groups. Finally, we identified TSPO as an independent predictor of esophageal cancer and validated its predictive capability. We also examined the association of TSPO expression with tumor cell stemness and its mutational status across various cancers. Experimentally, we performed clonogenic assays and CCK-8 assays to verify the enhanced proliferative capacity of tumor cells following TSPO overexpression. Furthermore, we validated the changes in TSPO expression levels using a mouse model of orthotopically induced esophageal cancer.

To characterize the cellular heterogeneity of esophageal squamous cell carcinoma (ESCC), we analyzed single-cell RNA sequencing data (GSE188900) from one normal sample and six tumor samples. UMAP visualization revealed a distinct separation between tumor cells and normal cells, highlighting significant transcriptional differences (**Figures 1B, C**). Unsupervised clustering identified 13 distinct cell populations (**Figure 1D**). Cell type annotation using SingleR determined major identities, including epithelial cells, endothelial cells, T cells, B cells, monocytes, smooth muscle cells, and tissue stem cells (**Figure 1E**). The enrichment of epithelial and immune cells in tumor samples indicated the presence of a complex microenvironment. Gene set enrichment analysis further confirmed the cell type specificity among the clusters, revealing distinct functional programs in epithelial, immune, and stromal cell populations (**Figure 1F**). These findings provide a comprehensive perspective on the cellular composition and heterogeneity of ESCC.

### Tumor stemness features in ESCC at the single-cell level

**Figure 2:**
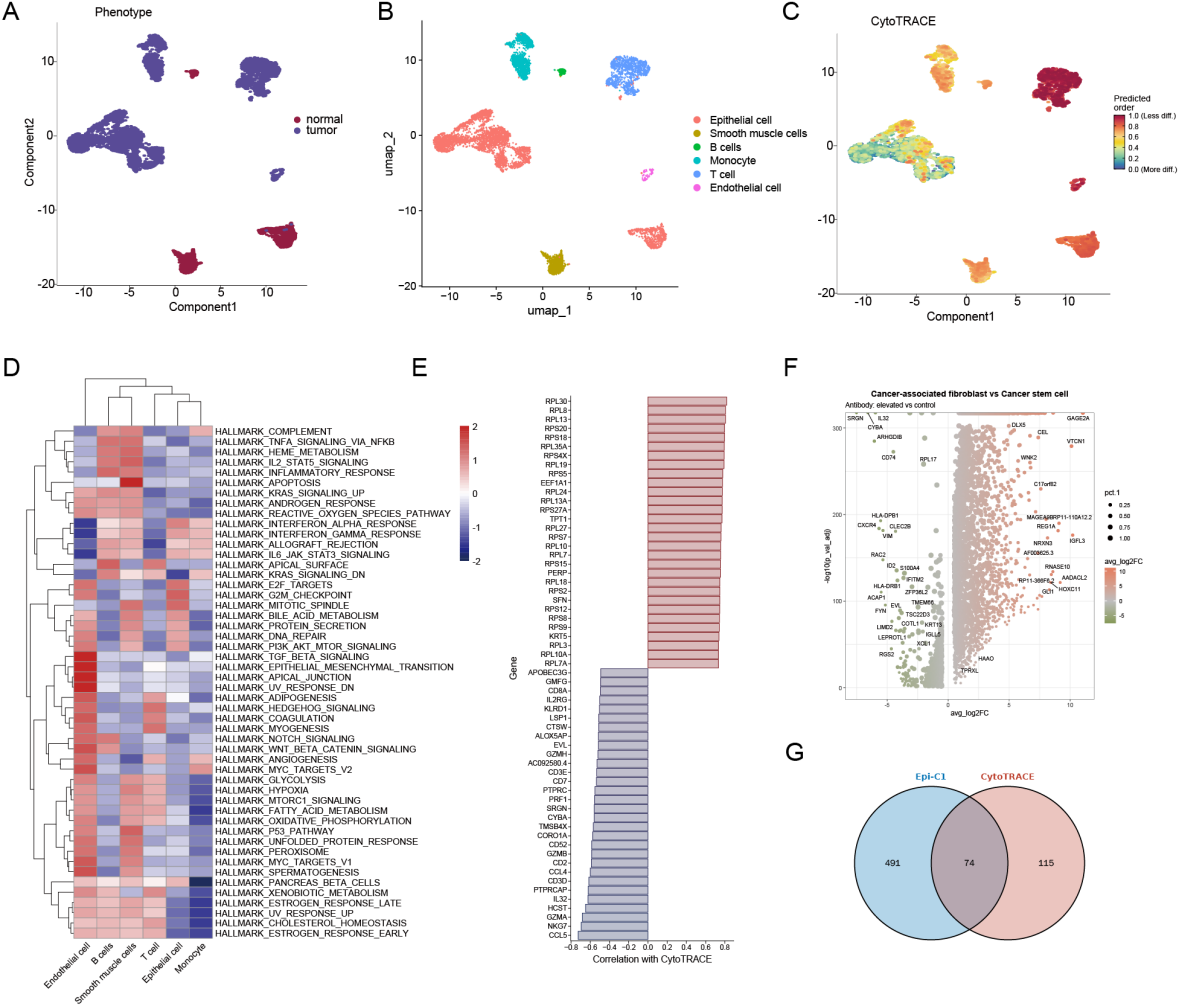
Tumor stemness features in ESCC at the single-cell level. **(A)** The UMAP plot displays the distribution of normal and tumor epithelial cells within ESCC samples. **(B)** UMAP visualization depicts annotated cell types, which include epithelial cells, T cells, B cells, monocytes, smooth muscle cells, and endothelial cells. **(C)** CytoTRACE analysis reveals the stemness scores of individual cells, with warmer colors indicating a higher potential for stemness. **(D)** A heatmap illustrates the enrichment of hallmark pathways across various cell types. **(E)** Bar plots represent genes that are positively and negatively correlated with CytoTRACE scores. **(F)** A volcano plot shows the differentially expressed genes between cancer-associated fibroblasts and cancer stem-like epithelial cells. **(G)** The Venn diagram illustrates the overlap between Epi-C1 marker genes and CytoTRACE-associated genes.

To further characterize the stemness features of epithelial cells within the ESCA tumor microenvironment, we employed multiple computational analysis methods that integrate phenotype, cell type identity, and stemness prediction scores (**Figures 2A–C**). UMAP visualization revealed distinct clusters of cells based on phenotype (normal vs. tumor, **Figure 2A**), annotated cell types (**Figure 2B**), and CytoTRACE-predicted stemness scores (**Figure 2C**). Notably, tumor-derived epithelial cells exhibited higher CytoTRACE scores compared to those from normal tissues, indicating enhanced developmental potential and putative stem cell-like characteristics (**Figure 2D**). GSVA pathway enrichment analysis of six major cell types revealed that tumor epithelial cells were specifically enriched in hallmark pathways related to inflammation (TNF-α/NFκB signaling, IL6/JAK/STAT3 signaling), proliferation (E2F, MYC targets), and epithelial-mesenchymal transition (EMT), all closely linked to cancer stemness (**Figure 2E**). Correlation analysis between gene expression and CytoTRACE scores identified genes positively or negatively associated with stemness. Ribosomal protein genes (e.g., RPL13, RPLP1, RPS12) exhibited strong positive correlations, consistent with previous reports implicating ribosomal biogenesis in stem-like states (**Figure 2F**). Differential expression analysis between cancer-associated fibroblasts (CAFs) and cancer stem-like epithelial cells revealed a unique gene expression profile, highlighting potential interactions and molecular differences between the stromal and stem-like tumor compartments (**Figure 2G**). Venn diagram analysis identified 74 overlapping genes between the Epi-C1 cluster and CytoTRACE-derived stemness-related genes, supporting the robustness of stemness signal identification across independent analytical strategies.

### A prognostic model based on stemness-related genes in ESCC

**Figure 3:**
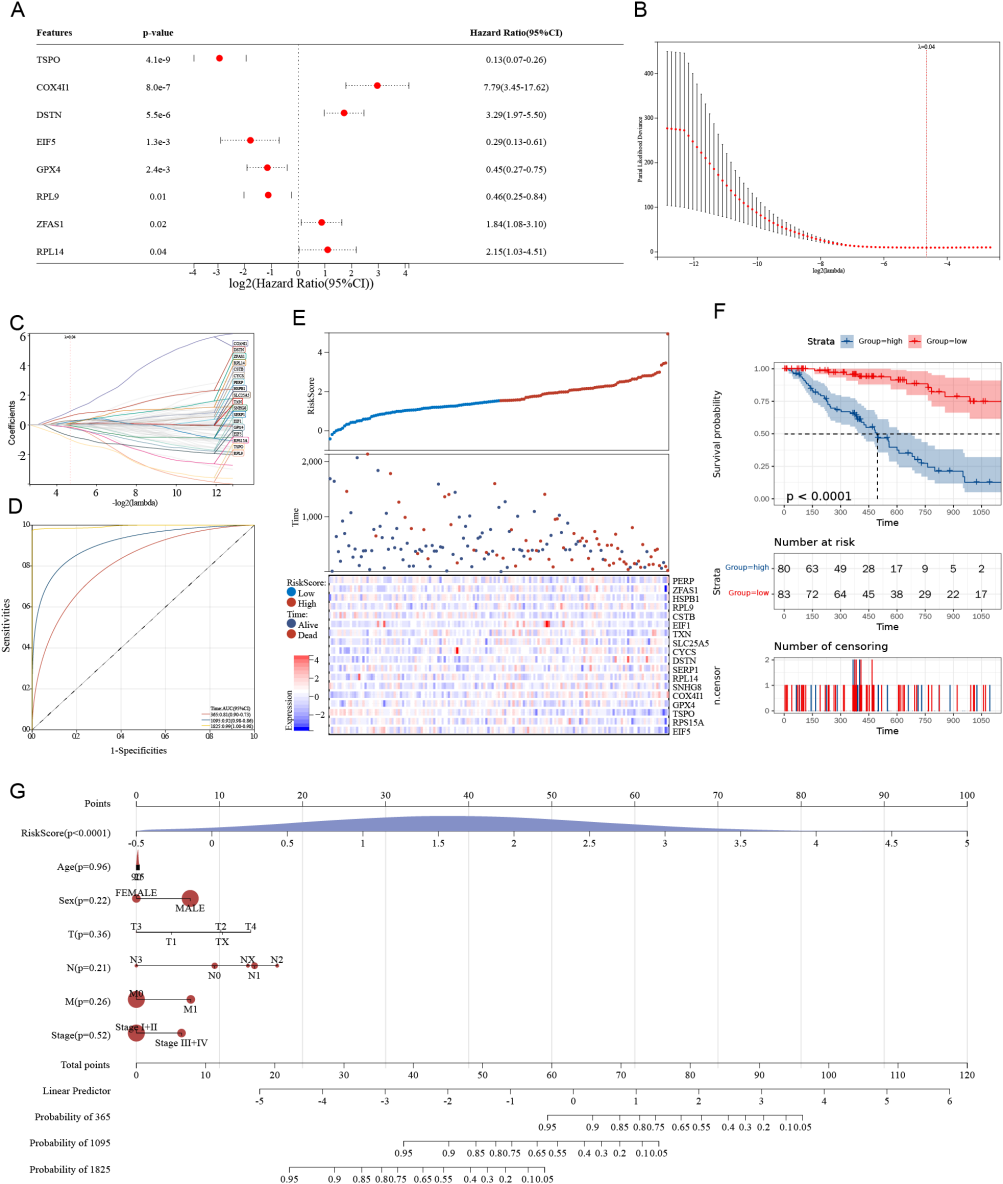
A prognostic model based on stemness-related genes in ESCC. **(A)** The forest plot displays the results of univariate Cox regression, highlighting hazard ratios and their significance for eight stemness-related genes. **(B)** The LASSO regression coefficient profile plot is presented across various log(λ) values. **(C)** The cross-validation curve is utilized to select the optimal λ for the LASSO model. **(D)** The ROC curve demonstrates the predictive accuracy of the risk model at 1, 3, and 5 years. **(E)** The distribution of risk scores, survival status, and a heatmap of gene expression are shown for high- and low-risk groups. **(F)** Kaplan-Meier survival analysis is conducted to compare overall survival between high-risk and low-risk groups. **(G)** A nomogram is provided, integrating risk scores and clinical parameters to predict survival probabilities at 1, 3, and 5 years.

To construct a prognostic model based on stemness-related genes in esophageal cancer (ESCA), univariate Cox regression identified eight significant genes, including TSPO, COX4I1, and DSTN. Notably, TSPO exhibited a strong protective effect, with a hazard ratio (HR) of 0.13 and a p-value of less than 0.001 (**Figure 3A**). LASSO regression further refined the feature selection process (**Figure 3B**), and the TSCMS risk score was calculated using the coefficients of the selected genes (**Figure 3C**). Receiver operating characteristic (ROC) analysis demonstrated strong predictive performance for 1-, 3-, and 5-year survival, with area under the curve (AUC) values of 0.82, 0.84, and 0.85, respectively (**Figure 3D**). In the validation cohorts GSE53625 and GSE19417, our risk model demonstrated moderate predictive performance. Time-dependent ROC analysis for GSE53625 yielded AUCs of 0.65, 0.55, and 0.50 for 1-, 3-, and 5-year overall survival, respectively (**Supplementary Figure 1A**). Similarly, in GSE19417, the AUCs were 0.58, 0.57, and 0.63 for 1-, 3-, and 5-year survival, respectively (**Supplementary Figure 1B**). These results suggest that the prognostic accuracy of the model may diminish over time. Patients were stratified into high-risk and low-risk groups based on the median risk score (**Figure 3E**). Kaplan-Meier analysis revealed significantly poorer survival outcomes in high-risk individuals, with a p-value of less than 0.0001 (**Figure 3F**). Kaplan-Meier survival analysis in the validation cohort GSE53625 showed that high-risk patients had significantly poorer overall survival compared to the low-risk group (*P* = 0,05, HR =1.49, **Supplementary Figure 1A**), Kaplan-Meier survival analysis in the validation cohort GSE19417 showed that high-risk patients had significantly poorer overall survival compared to the low-risk group (*P* = 0,04, HR =0.57, **Supplementary Figure 1B**),confirming the prognostic value of the model. Finally, nomogram analysis, which incorporated clinical features alongside risk scores, demonstrated that the TSCMS model possesses robust predictive value, with the risk score identified as the most influential variable for long-term survival prediction (**Figure 3G**). These findings underscore the potential clinical utility of the TSCMS model in stratifying ESCA patients and guiding individualized prognostic assessments.

### Functional enrichment analysis based on the TSCMS model

**Figure 4:**
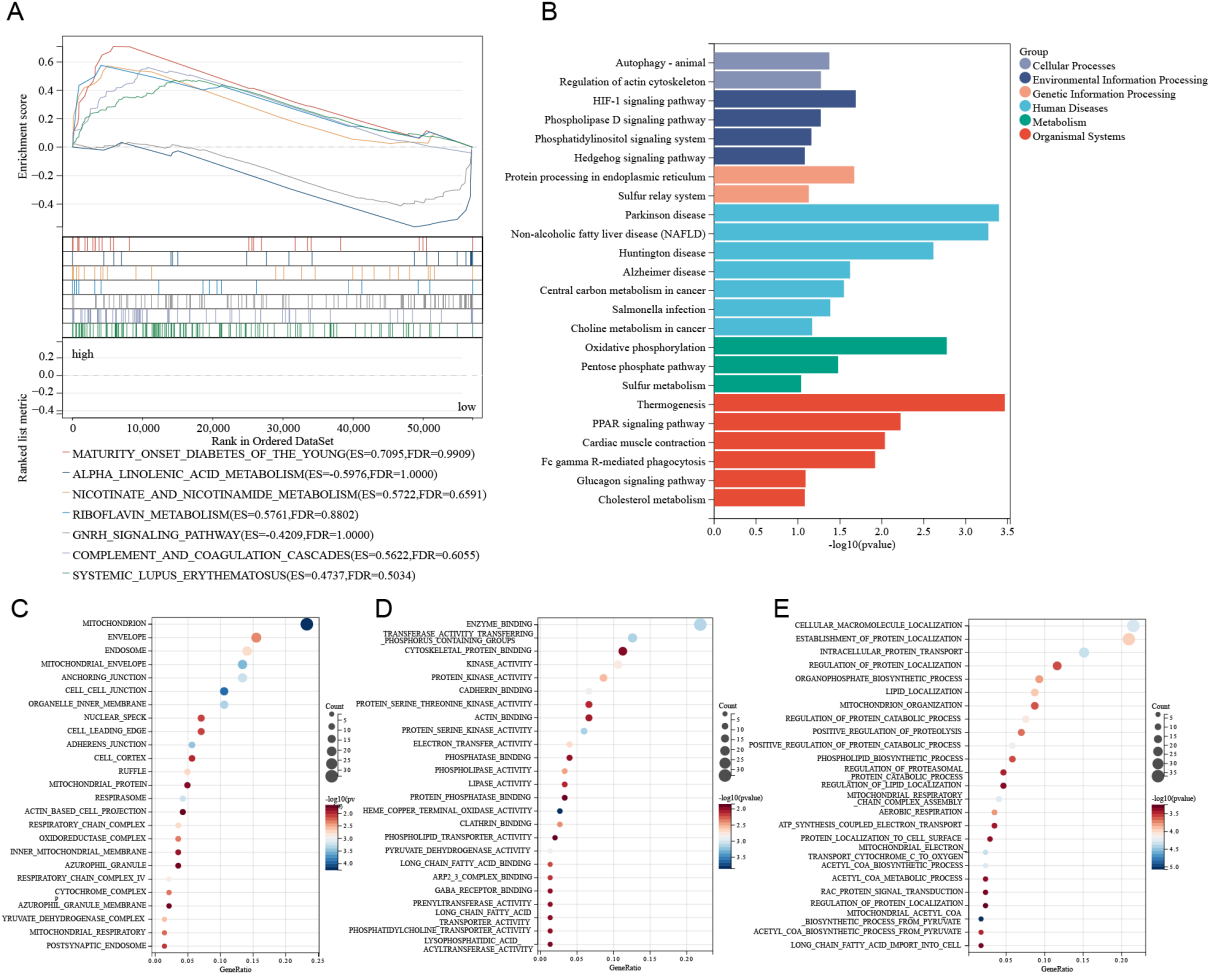
Functional enrichment analysis based on the TSCMS model. **(A)** The GSEA enrichment plot illustrates significantly enriched pathways between high-risk and low-risk groups in the TSCMS model. **(B)** The KEGG pathway enrichment analysis results are presented in bar graph format, categorized by functional groups. **(C–E)** GO enrichment analysis of differentially expressed genes between high-risk and low-risk groups in the TSCMS model.

To further investigate the functional roles of stemness-related genes in the TSCMS model, we performed Gene Set Enrichment Analysis (GSEA) and functional enrichment analyses on high- and low-risk groups. GSEA revealed that the high stemness expression group was not significantly enriched in classical pathways (**Figure 4A**), indicating a distinct biological context. KEGG pathway analysis revealed that these genes are primarily involved in metabolic and signaling pathways, including cholesterol metabolism, sulfur metabolism, glucose homeostasis, and PPAR signaling pathways (**Figure 4B**). Additionally, Gene Ontology (GO) analysis demonstrated, from three perspectives—molecular function (**Figure 4C**), cellular component (**Figure 4D**), and biological process (**Figure 4E**) that stemness-related genes are concentrated in key biological processes such as mitochondrial function, protein localization and transport, energy metabolism, and lipid metabolism regulation. These results indicate that the TSCMS gene cluster is not only involved in the maintenance of tumor stemness but may also regulate tumor metabolic reprogramming, highlighting its potential functional diversity and therapeutic target value in ESCA.

### Results of drug sensitivity analysis in the high-risk and low-risk groups

**Figure 5:**
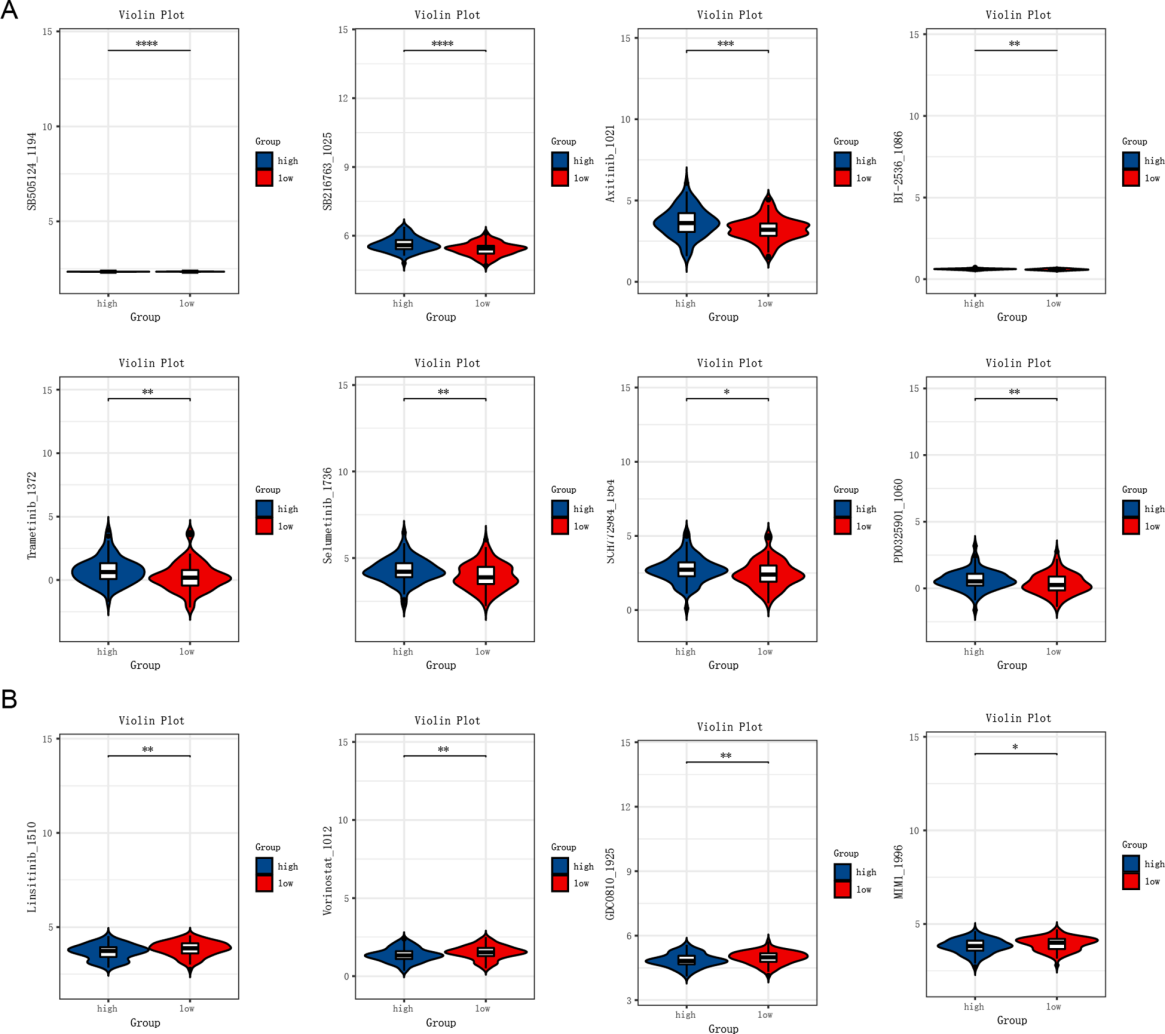
Results of drug sensitivity analysis in the high-risk and low-risk groups. **(A)** Drugs to which the high-risk group is more sensitive. **(B)** Drugs to which the low-risk group is more sensitive. Differences in drug response between high- and low-risk groups were compared using the Wilcoxon rank-sum test. * *P* < 0.05 statistically significant; ** *P* < 0.01 very significant; *** *P* < 0.001 highly significant; **** *P* < 0.0001 extremely significant.

To investigate the relationship between TSCMS scores and drug response, we systematically assessed differences in sensitivity to various small-molecule drugs between high- and low-TSCMS groups based on predicted half-maximal inhibitory concentration (IC50) values (**Figures 5A, B**). The results indicated that the high TSCMS score group exhibited lower IC50 values across multiple targeted drugs, signifying greater sensitivity to these agents. The IC50 values of drugs such as SB505124 (a TGF-β inhibitor), SB216763 (a GSK-3 inhibitor), Akt inhibitor VIII, and BI.2536 (a PLK1 inhibitor) were significantly reduced in the high-score group (P < 0.001), suggesting that the stemness state may enhance the response to these signaling pathway inhibitors. Furthermore, the high-score group demonstrated increased sensitivity to commonly used clinical drugs, including Tamoxifen (an estrogen receptor modulator), Sunitinib (a multi-target tyrosine kinase inhibitor), and PHA665752 (a MET inhibitor). Meanwhile, the low TSCMS score group exhibited lower IC50 values for certain drugs, indicating that it might mediate drug responses through different mechanisms. This finding suggests that the TSCMS score can not only effectively distinguish patients’ prognostic risks but may also serve as a crucial tool for guiding individualized drug selection. In summary, this model is anticipated to play a key role in the precision treatment of esophageal cancer, particularly in aiding the development of stemness state-driven therapeutic strategies, thereby demonstrating potential clinical value.

### Correlation between TSPO expression and various aspects of tumor biology, including stemness, heterogeneity, and gene mutations in cancer

**Figure 6:**
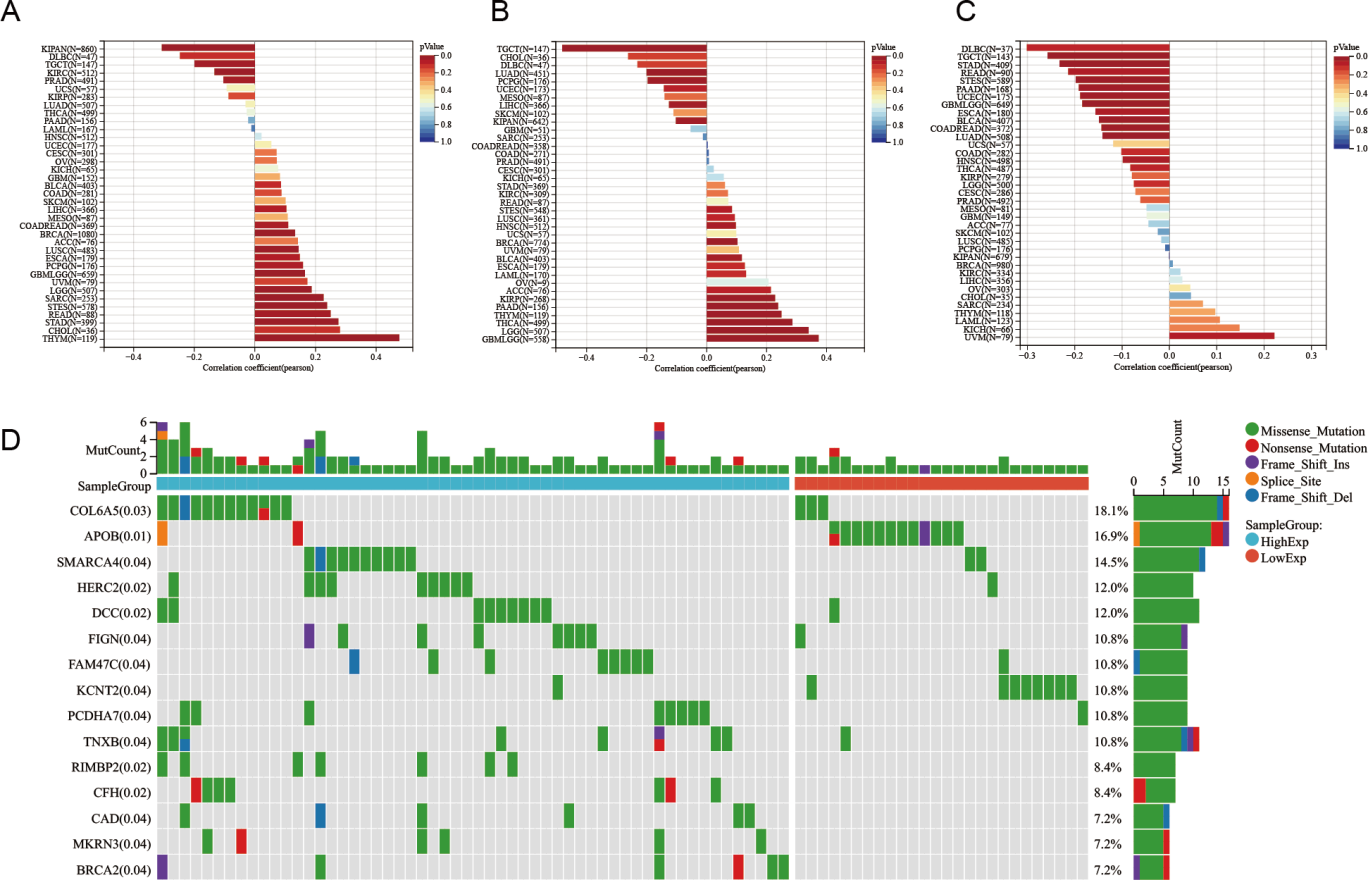
Correlation between TSPO expression and various aspects of tumor biology, including stemness, heterogeneity, and gene mutations in cancer. **(A)** The correlation between TSPO expression and EREG.EXPss stemness scores, as derived from RNA expression profiles, is presented. **(B)** The relationship between TSPO expression and DNAss stemness scores, which are based on DNA methylation signatures, is also depicted. **(C)** Furthermore, the correlation between TSPO expression and tumor heterogeneity is quantified using the Mutant-Allele Tumor Heterogeneity score, revealing a significant negative correlation specifically in esophageal cancer. **(D)** A waterfall plot is included to showcase the gene mutation profiles of 181 esophageal cancer samples, categorized into high and low TSPO expression groups. Notably, COL6A5 emerged as the most significantly mutated gene, followed by SMARCA4, HERC2, DCC, FIGN, and others, with mutation types distinctly color-coded.

We obtained six tumor stemness indices derived from mRNA expression and methylation signatures, based on prior research (Machine Learning Identifies Stemness Features Associated with Oncogenic Dedifferentiation). These indices include EREG.EXPss, which reflects RNA expression influenced by epigenetic regulation (103 genes) (**Figure 6A**), and DNAss, which represents DNA methylation based on stem cell characteristic probes (219 probes) (**Figure 6B**). We used these indices to evaluate the relationship between TSPO expression and tumor cell stemness. The results showed a strong correlation between TSPO expression and stemness features across various cancer cells. Mutant-Allele Tumor Heterogeneity quantifies the deviation in the distribution of mutant allele frequencies (MAF) at tumor-specific variant sites, reflecting how much the MAF distribution diverges from the overall sample. Higher Mutant-Allele Tumor Heterogeneity values indicate increased tumor heterogeneity. The analysis of tumor heterogeneity revealed a negative correlation between TSPO expression and the heterogeneity of various cancer tumor cells (**Figure 6C**), particularly in esophageal cancer. Consequently, we further investigated the sequencing results of esophageal cancer, which included a total of 181 mutation-tested samples. The chi-square test was employed to assess the differences in gene mutation frequencies among the sample groups. The results indicated that mutations in the collagen type VI alpha 5 chain (COL6A5) were the most significant. Additionally, mutations in genes such as SMARCA4, HERC2, DCC, FIGN, FAM47C (STBD1), and KCNT2 were also notably significant (**Figure 6D**). These findings suggest that TSPO expression is closely associated with cancer progression.

### Correlation between TSPO expression and the immune microenvironment in ESCC

**Figure 7:**
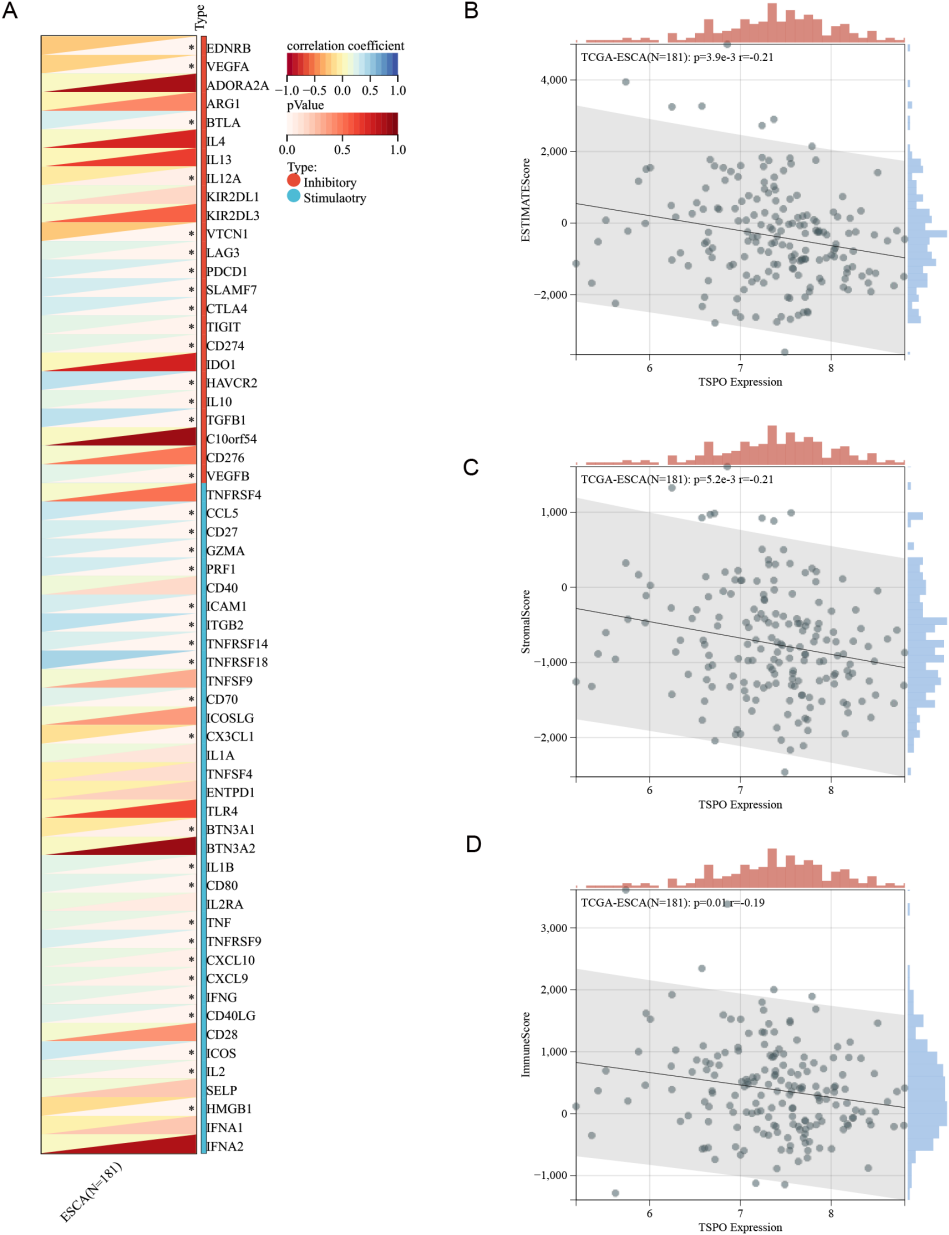
Correlation between TSPO expression and the immune microenvironment in ESCC. **(A)** The correlation heatmap displays the relationship between TSPO and immune checkpoint genes, encompassing both stimulatory and inhibitory molecules, within the TCGA-ESCA cohort (n = 181). **(B–D)** Scatter plots depict the correlation between TSPO expression and ESTIMATEScore **(B)**, StromalScore **(C)**, and ImmuneScore **(D)**. The Spearman correlation coefficients and corresponding p-values are provided.

To elucidate the association between key genes of TSCMS and the immune microenvironment, we further analyzed the immune-related characteristics of the key prognostic gene TSPO (**Figure 7**). **Figure 7A** shows the correlation between TSPO expression and several immune checkpoint molecules, revealing a significant negative association between TSPO and multiple inhibitory checkpoints (e.g., PDCD1, CTLA4, TIGIT, HAVCR2) and stimulatory (e.g., CD27, CD40, ICOS, TNFRSFs) immune factors, suggesting that TSPO may participate in tumor immune escape by regulating immune suppressive pathways. Furthermore, we evaluated the relationship between TSPO expression and tumor microenvironment (TME) scores. Correlation scatter plots indicated that TSPO expression levels were negatively correlated with Estimation of Stromal and Immune cells in Malignant Tumor tissues using Expression data (ESTIMATE) scores (**Figure 7B**), StromalScore (**Figure 7C**), and ImmuneScore (**Figure 7D**), with correlation coefficients of -0.21, -0.21, and -0.19, respectively, all of which were statistically significant (P < 0.05). These results indicate that high TSPO expression may be associated with lower levels of immune infiltration, further validating its potential inhibitory role in the immune microenvironment. In summary, as a key constituent gene of the TSCMS score, TSPO may influence the tumor immune microenvironment by inhibiting immune cell infiltration and downregulating the expression of immune activity-related factors, thereby participating in the progression and prognosis regulation of esophageal cancer. This discovery provides a theoretical basis for subsequent TSPO-targeted immunotherapy.

### Downregulation of TSPO in ESCC and its role in suppressing tumor cell proliferation

**Figure 8:**
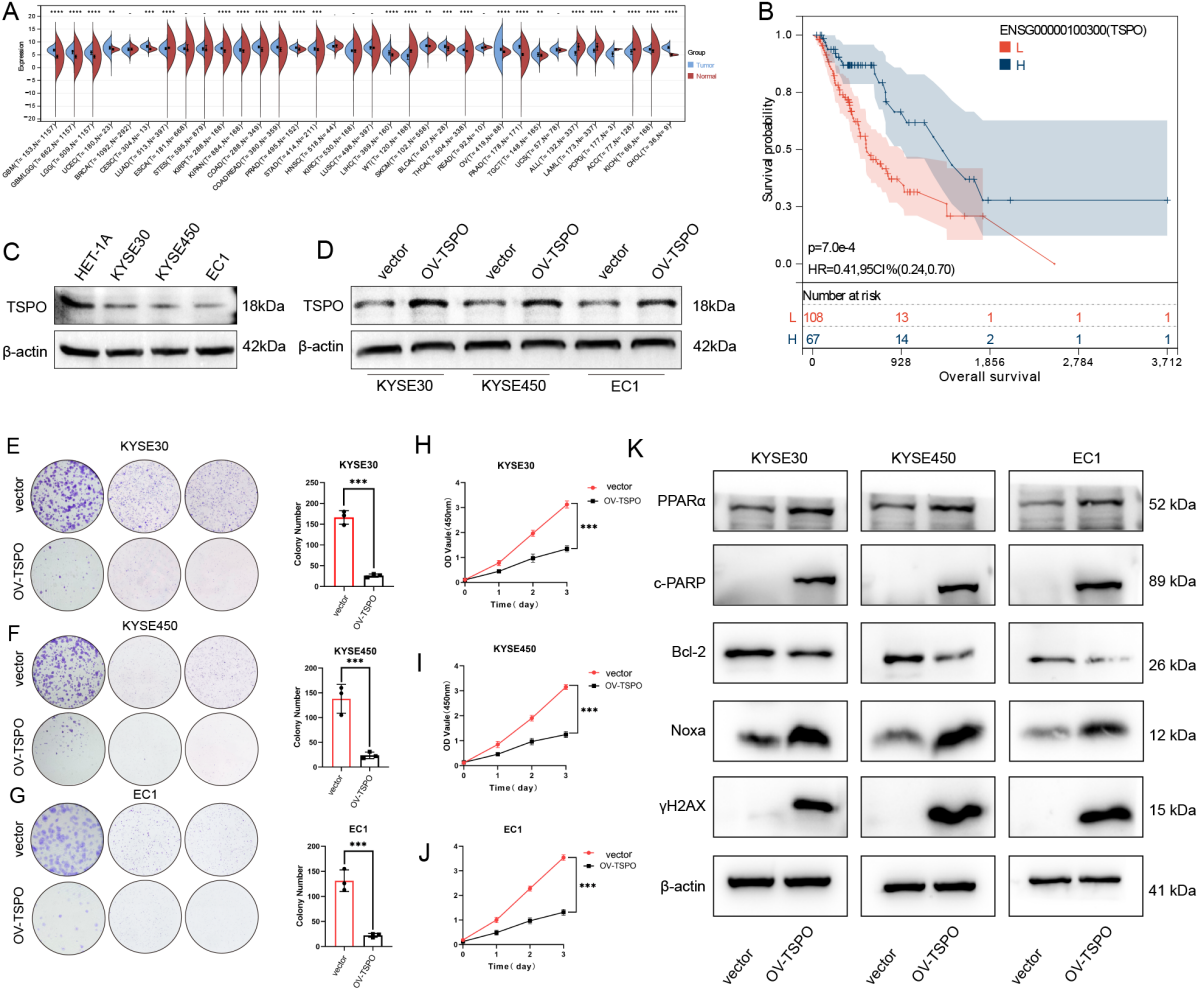
Downregulation of TSPO in ESCC and its role in suppressing tumor cell proliferation. **(A)** The expression of TSPO across various cancer types, as analyzed from the TCGA and GTEx datasets, reveals a notable reduction in ESCC, which is highlighted for emphasis. **(B)** The Kaplan–Meier survival curve demonstrates that lower TSPO expression is associated with poorer overall survival outcomes in the TCGA-ESCA cohort of ESCC patients. **(C)** Western blot analysis reveals TSPO expression levels in normal esophageal epithelial cells (HET-1A) compared to ESCC cell lines (KYSE30, KYSE450, EC1). **(D)** Western blot results confirm the efficiency of TSPO overexpression (OV-TSPO) in ESCC cells. **(E–G)** Colony formation assays conducted in KYSE30 **(E)**, KYSE450 **(F)**, and EC1 **(G)** cells indicate that TSPO overexpression significantly inhibits clonogenic ability. **(H–J)** CCK-8 assays demonstrate a reduction in proliferation in TSPO-overexpressing KYSE30 **(H)**, KYSE450 **(I)**, and EC1 **(J)** cells, with statistical significance indicated as ***P < 0.001. **(K)** Western blot analysis of key apoptotic and DNA damage-related proteins (PPARα, c-PARP, Bcl-2, Noxa, and γH2AX) in TSPO-overexpressing ESCC cells. β-actin was used as the loading control. All data were representative of at least three independent experiments (n = 3; error bar, SD). Statistical significance: *P* < 0.05 (*), *P *< 0.01 (**), *P* < 0.001 (**), *P* < 0.0001 (****).

To further validate the expression characteristics and functional roles of the key gene TSPO in esophageal cancer, we conducted a systematic expression analysis and functional experimental validation (**Figure 8**). As illustrated in **Figure 8A**, the expression level of TSPO exhibited significant variation across multiple cancer types, particularly in esophageal cancer (ESCA) tissues, where its expression was markedly lower than in normal tissues (*P* < 0.0001). Kaplan–Meier survival analysis based on the TCGA database further indicated that high expression of TSPO was significantly associated with longer overall survival (OS) (**Figure 8B**, P = 7.0e-4, HR = 0.41, 95% CI = 0.24–0.70), suggesting that TSPO may possess tumor-suppressive functions. Furthermore, we validated our findings using the GEO dataset GSE53625, which showed that patients with high TSPO expression had significantly better overall survival than those with low TSPO expression(**Supplementary Figure 1C**). At the protein level, Western blot analysis revealed that TSPO expression was higher in the normal esophageal epithelial cell line HEF1A, but significantly downregulated in ESCA cell lines KYSE30, KYSE450, and EC1 (**Figure 8C**). We subsequently constructed TSPO-overexpressing (OV-TSPO) and control (vector) cell lines and validated their protein expression levels (**Figure 8D**). In addition, the overexpression efficiency of TSPO at the RNA level was validated, as shown in **Supplementary Figures 1D–F**. The results of the colony formation assay demonstrated that TSPO overexpression significantly inhibited the colony-forming ability in KYSE30 (**Figure 8E**), KYSE450 (**Figure 8F**), and EC1 (**Figure 8G**) cells, indicating its inhibitory effect on the proliferation of esophageal cancer cells (all *P* < 0.001). Meanwhile, the CCK-8 proliferation assay also confirmed that TSPO overexpression significantly inhibited the increase in cell viability compared to the control group (**Figures 8H–J**, *P* < 0.001), with consistent results across multiple cell lines. In summary, TSPO is expressed at low levels in esophageal cancer, and its high expression is associated with a favorable prognosis. Functional experiments have validated its significant tumor-suppressing activity. These findings suggest that TSPO may represent a potential prognostic biomarker and therapeutic target in esophageal cancer. Overexpression of TSPO significantly altered the expression of key proteins involved in apoptosis and DNA damage pathways. As shown in **Figure 8K**, TSPO overexpression led to increased levels of PPARα and cleaved PARP (c-PARP), decreased expression of the anti-apoptotic protein Bcl-2, and elevated expression of the pro-apoptotic protein Noxa in KYSE30, KYSE450, and EC1 cells. In addition, the expression of γH2AX, a marker of DNA double-strand breaks, was markedly upregulated, indicating enhanced DNA damage. These findings suggest that TSPO may promote apoptosis and DNA damage responses in ESCC cells, thereby suppressing tumor cell survival.

### Histological and immunohistochemical analysis of TSPO in human ESCC and adjacent normal tissues

**Figure 9:**
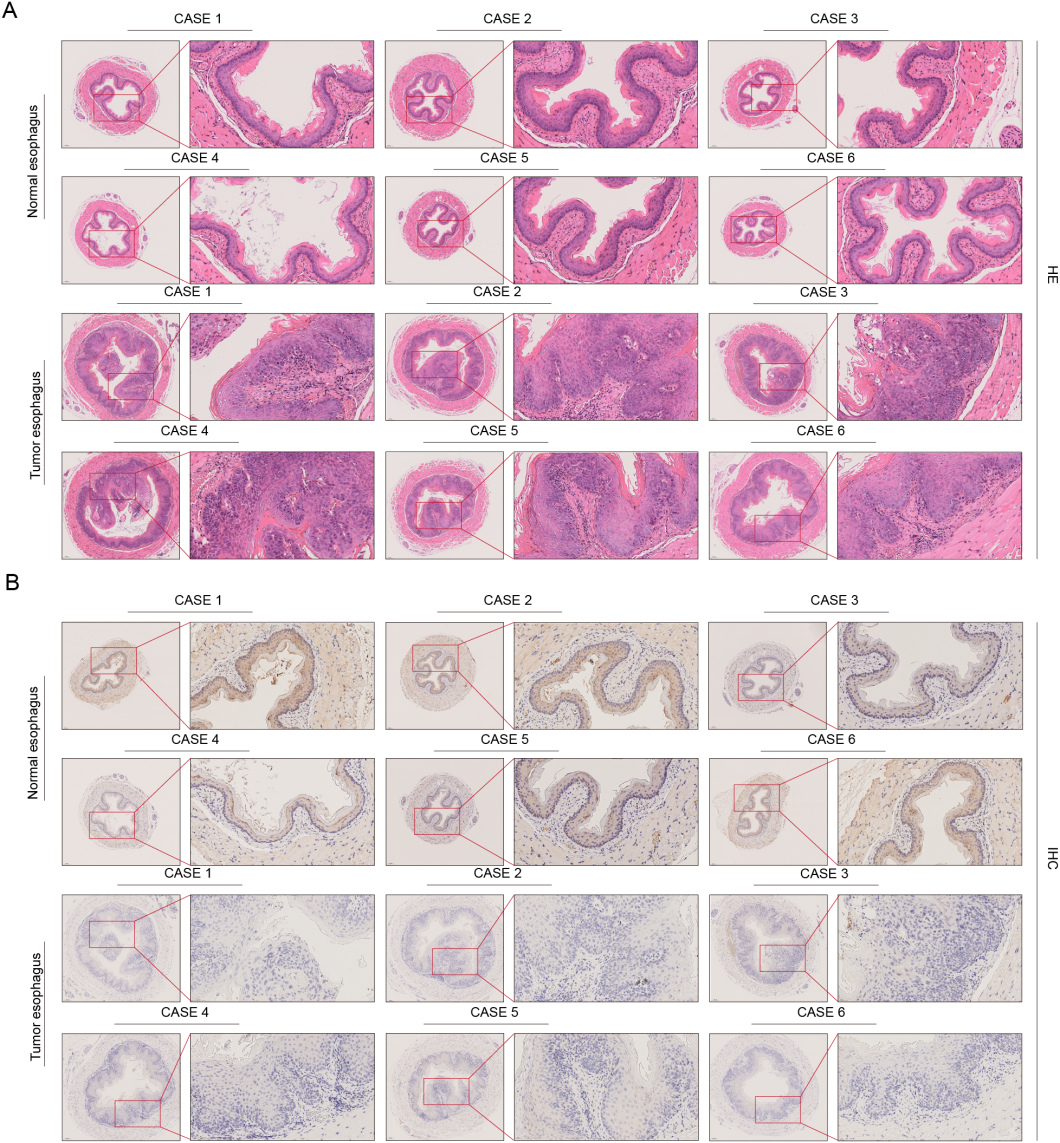
Histological and immunohistochemical analysis of TSPO in human ESCC and adjacent normal tissues. **(A)** Hematoxylin and eosin (H&E) staining of normal and tumor esophageal tissues from six ESCC patients illustrates the morphological differences between the two groups. **(B)** Immunohistochemistry (IHC) staining of TSPO within the same patient cohort demonstrates a significantly stronger expression of TSPO in normal esophageal epithelium compared to tumor tissues. Insets provide magnified views for detailed visualization.

To further validate the differential expression of TSPO in esophageal cancer tissues, we established a mouse model of esophageal carcinoma *in situ* and obtained esophageal tissues at 18 weeks, which were subsequently subjected to hematoxylin and eosin (HE) staining and immunohistochemical (IHC) staining analysis (**Figure 9**). **Figure 9A** presents the results of HE staining, where normal esophageal tissue displays a typical stratified squamous epithelial structure characterized by orderly cell arrangement and the absence of significant atypia. In contrast, the esophageal cancer tissue exhibits pronounced structural disorganization, disordered epithelial cell arrangement, prominent cellular atypia, and infiltration of cancer nests in certain areas, indicating typical tumor-like changes in the tissue. The IHC staining results shown in **Figure 9B** reveal that TSPO displays moderate to strong positive expression in normal esophageal tissues, primarily localized in the cytoplasm of epithelial cells, with uniformly distributed staining. In esophageal tumor tissues, TSPO expression significantly decreased, with most regions showing weak positive or negative staining and a notable reduction in staining intensity. This trend was consistent across six cases, further validating the downregulated expression characteristics of TSPO in esophageal cancer tissues. TSPO exhibited a gradual downregulation from normal to tumor tissues at the histological level, which, combined with the aforementioned protein level and functional experimental results, further supports the research foundation of TSPO as a potential tumor suppressor factor in esophageal cancer.

## Discussion

In the rapidly advancing field of biomedical research, advanced optimization and feature selection techniques have become powerful tools. Numerous studies have developed novel algorithms to improve the performance of diagnostic and prognostic models, thereby enhancing disease prediction accuracy and elucidating underlying biological mechanisms (17). While previous efforts have predominantly focused on constructing prognostic models for esophageal squamous cell carcinoma (ESCC) based on immune infiltration and cancer-associated fibroblasts (CAFs) (9, 18), our study shifts the focus to tumor stemness in ESCC. By integrating stemness-related gene signatures with single-cell RNA sequencing data, we identified distinct epithelial cell subpopulations exhibiting stem-like characteristics within the tumor microenvironment. This led to the development of a novel prognostic risk model, termed TSCMS (Tumor Stemness-Cell-based Molecular Score). Notably, TSCMS outperforms traditional models by incorporating both molecular and clinical parameters, offering improved predictive accuracy for patient survival and providing valuable guidance for personalized clinical decision-making.

In our study, we identified the genes TSPO, COX4I1, DSTN, EIF5, GPX4, RPL9, ZFAS1, and RPL14 as potentially critical players in the progression of esophageal squamous cell carcinoma. TSPO and COX4I1 are mitochondrial-associated proteins that participate in metabolic processes and oxidative phosphorylation, potentially contributing to the reprogramming of tumor energy metabolism (19–21). DSTN is suggested to enhance cancer cell migration and invasion by modulating actin dynamics (22). EIF5 facilitates protein synthesis and cellular proliferation, aligning with its established oncogenic roles in various cancers (23–25). GPX4, an inhibitor of ferroptosis, aids in cell survival under oxidative stress, which may lead to therapeutic resistance (26). Ribosomal proteins RPL9 and RPL14 not only play essential roles in translation but also impact the cell cycle and apoptosis, indicating their broader implications in tumor biology (27, 28). Additionally, ZFAS1, a long non-coding RNA, has been associated with increased proliferative capacity and stemness (28, 29). Collectively, these genes are involved in key pathways including metabolism, redox regulation, protein synthesis, and cellular plasticity, highlighting their potential as prognostic biomarkers and therapeutic targets in ESCC.

In the TCGA training dataset, the Tumor Subtype Classification and Molecular Signature (TSCMS) model revealed a significant difference in median survival between high- and low-risk patients, exceeding five years. Additionally, the model demonstrated robust predictive accuracy for 1-, 3-, and 5-year survival, with AUC values consistently above 0.7 across all three cohorts. It is noteworthy that, compared to the risk model developed by Zhou et al (30)., which centers on the CDKL3-related autophagy (CrA) risk score, and the model proposed by Cai et al (31)., which focuses on LAG3-related immunity and prognosis, our TSCMS demonstrates higher accuracy in predicting patient survival rates. This highlights the robust predictive capability of TSCMS in forecasting patient survival rates.

Among the genes identified in our model, TSPO (translocator protein) has emerged as a significant mitochondrial biomarker implicated in cancer biology. TSPO is known to regulate mitochondrial membrane permeability, oxidative stress responses, and apoptosis (32). Previous studies have reported dysregulated TSPO expression across various malignancies, including breast cancer (33–35), head and neck squamous cell carcinoma (36), and oral squamous cell carcinoma (37, 38), where its expression levels correlate with tumor aggressiveness and patient prognosis. Although direct studies on TSPO in ESCC remain limited, its established involvement in mitochondrial dysfunction and redox regulation indicates that it may also influence tumor progression and therapeutic responses in ESCC (19). These findings underscore the relevance of TSPO as both a mechanistic contributor and a potential biomarker in ESCC, warranting further investigation. In this study, we employed both cell and animal experiments to investigate the role of TSPO in the progression of esophageal cancer. The results demonstrated that a reduction in TSPO expression significantly enhanced cell proliferation. Furthermore, in the mouse orthotopic esophageal cancer model, we observed a notable decrease in TSPO expression. These findings indicate that TSPO may serve as a promising target for cancer suppression.

However, this study has several limitations. First, although the TSCMS model demonstrated strong predictive performance, its clinical utility requires independent validation in larger, prospective ESCA cohorts. The current sample size may limit the generalizability of our findings, and future studies should address this to confirm the model’s robustness. Second, while key pathways associated with TSPO and cancer stemness were identified, direct experimental validation is lacking. For instance, although TSPO overexpression experiments were conducted, we did not perform TSPO knockdown studies in ESCC cell lines, as TSPO expression is generally low in the majority of ESCC models. This limited the feasibility and interpretability of loss-of-function assays. Nevertheless, future studies utilizing patient-derived models or engineered systems with high TSPO expression are warranted to determine whether TSPO depletion might enhance tumor growth or stemness. Further in-depth mechanistic investigations—including *in vivo* studies—are also needed to elucidate the precise molecular role of TSPO in tumor progression and stem cell regulation in ESCA. Lastly, although the TSCMS model shows promise in predicting prognosis, additional clinical trials and mechanistic studies are essential to evaluate its utility in guiding therapeutic decision-making, particularly in the context of immunotherapy. These validations are critical for advancing the clinical applicability and biological relevance of our findings.

While our findings highlight the potential therapeutic relevance of TSPO in ESCA, further studies are needed to refine targeting strategies and identify the most effective approaches. The incorporation of advanced optimization and feature selection techniques may enhance analytical rigor and improve the accuracy of identifying molecular interactions involved in tumor progression. Clinically, targeting TSPO via specific inhibitors or RNA interference holds promise for suppressing ESCA development. Moreover, integrating the TSCMS model into diagnostic workflows could enable early identification of high-risk patients, facilitating timely and personalized therapeutic interventions.

## Conclusion

In summary, this study highlights the prognostic value of the TSCMS model in ESCA and identifies TSPO as a potential therapeutic target. Nonetheless, further research is required to validate its clinical relevance and explore its therapeutic potential.

## Data Availability

Publicly available datasets were analyzed in this study. This data can be found here: https://www.ncbi.nlm.nih.gov/geo/query/acc.cgi?acc=GSE21257; https://www.ncbi.nlm.nih.gov/geo/query/acc.cgi; https://www.ncbi.nlm.nih.gov/geo/query/acc.cgi?acc=GSE162454; https://www.ncbi.nlm.nih.gov/geo/query/acc.cgi?acc=GSE152048.

## Supplementary material

Supplementary Figure 1 External validation of the prognostic model and TSPO overexpression efficiency. **(A, B)** Time-dependent ROC curves for the risk model in the GSE53625 and GSE19417 validation cohort, with AUCs of 0.65, 0.55, and 0.50 for 1-, 3-, and 5-year overall survival, respectively. Kaplan–Meier survival curves showing that patients in the high-risk group had significantly poorer overall survival than those in the low-risk group in the GSE53625 cohort. **(C)** Kaplan-Meier analysis of the GSE53625 cohort indicating that patients with high TSPO expression had significantly better overall survival compared to those with low TSPO expression. **(D–F)** Validation of TSPO overexpression efficiency at the RNA level in ESCC cell lines (KYSE30, KYSE450, and EC1), as determined by quantitative real-time PCR (qRT-PCR).
