# Supplementary figures and images for: Integration of single-cell and bulk RNA sequencing to identify unique tumor stem cells and construct novel prognostic markers for assessing ESCA prognosis and drug sensitivity
Source: Front Oncol. 2025 Aug 27;15:1649877. doi: 10.3389/fonc.2025.1649877 (PMC12421627; doi:10.3389/fonc.2025.1649877)

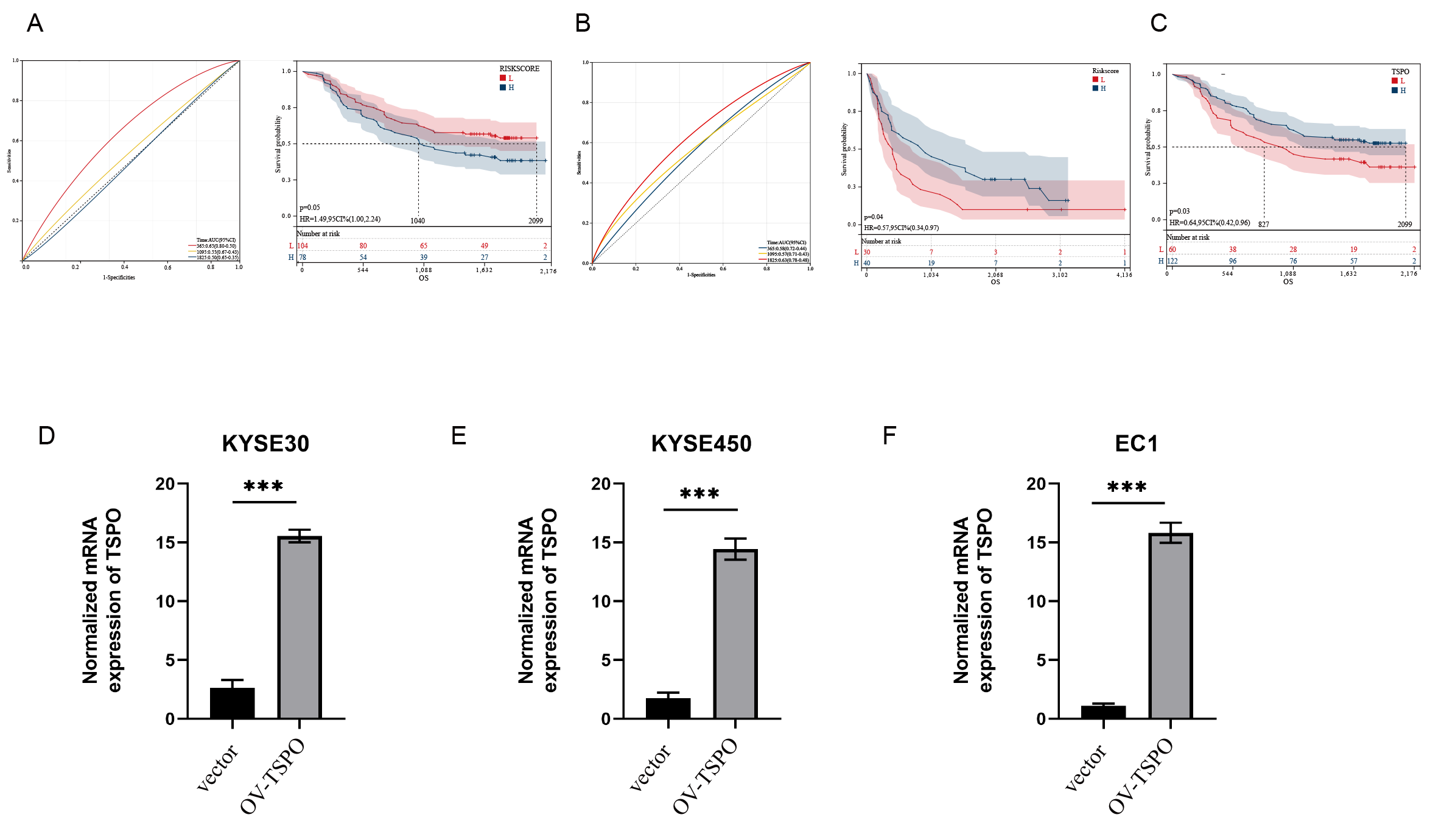

Supplement: Supplementary Figure 1 — External validation of the prognostic model and TSPO overexpression efficiency. (A, B) Time-dependent ROC curves for the risk model in the GSE53625 and GSE19417 validation cohort, with AUCs of 0.65, 0.55, and 0.50 for 1-, 3-, and 5-year overall survival, respectively. Kaplan–Meier survival curves showing that patients in the high-risk group had significantly poorer overall survival than those in the low-risk group in the GSE53625 cohort. (C) Kaplan-Meier analysis of the GSE53625 cohort indicating that patients with high TSPO expression had significantly better overall survival compared to those with low TSPO expression. (D–F) Validation of TSPO overexpression efficiency at the RNA level in ESCC cell lines (KYSE30, KYSE450, and EC1), as determined by quantitative real-time PCR (qRT-PCR). [file Image1.tif]
